# Hypoxia-inducible *APCDD1L-AS1* promotes osimertinib resistance by stabilising DLST to drive tricarboxylic acid cycle in lung adenocarcinoma

**DOI:** 10.1186/s13046-025-03462-z

**Published:** 2025-07-09

**Authors:** Quanli Zhang, Ye Shen, Yuru Che, Lili Jia, Xiang Xiao, Hao Xu, Chi Su, Kemin Sun, Limin Zheng, Jiawen Xu, Jingwen Hu, Chaofeng Zhang, Dihan Zhu, Ming Li

**Affiliations:** 1 School of Life Science and Technology, China Pharmaceutical University, 639 Longmian Avenue, Nanjing, 210009 Jiangsu P. R. China; 2 Department of Thoracic Surgery, the Affiliated Cancer Hospital of Nanjing Medical University & Jiangsu Cancer Hospital & Jiangsu Institute of Cancer Research, Jiangsu Key Laboratory of Innovative Cancer Diagnosis & Therapeutics, Collaborative Innovation Center for Cancer Personalized Medicine, Nanjing, 210009 Jiangsu P. R. China; 3 State Key Laboratory of Natural Medicines, School of Traditional Chinese Pharmacy, China Pharmaceutical University, 639 Longmian Avenue, Nanjing, 210009 Jiangsu P. R. China

**Keywords:** Osimertinib-resistant, Hypoxia, Tricarboxylic acid cycle, Lung adenocarcinoma, Long non-coding RNAs

## Abstract

Acquired resistance is unavoidable in lung adenocarcinoma (LUAD) treated with osimertinib, however, the underlying mechanisms remain largely unknown. Here, we report that the long non-coding RNA (lncRNA) APCDD1L-AS1 is upregulated in osimertinib-resistant LUAD tissues and cells and is associated with short survival of osimertinib-resistant LUAD patients. Our data showed that APCDD1L-AS1 upregulation is an independent risk factor for overall survival in patients with osimertinib-resistant LUAD. APCDD1L-AS1 knockdown enhanced osimertinib sensitivity both in vitro and in vivo, whereas APCDD1L-AS1 overexpression promoted osimertinib resistance. Mechanistically, APCDD1L-AS1 accelerates the tricarboxylic acid (TCA) cycle by forming complexes and maintaining the stability of dihydrolipoamide S-succinyltransferase (DLST), which inhibits the ubiquitination and degradation of DLST. Moreover, we demonstrate that hypoxia-inducible factor (HIF)-1α transcriptionally activates APCDD1L-AS1 by binding to the APCDD1L-AS1 promoter region under hypoxic conditions. Overall, our data confirm that APCDD1L-AS1 is upregulated by hypoxia-induced HIF-1α, which drives the TCA cycle by stabilising DLST to further promote osimertinib resistance in LUAD. Our findings provide new insights into the role of HIF-1α/APCDD1L-AS1/DLST axis-related reprogramming of hypoxia and the TCA balance in conferring osimertinib resistance in LUAD and confirm the therapeutic potential for targeting the APCDD1L-AS1.

**Supplementary Information:**

The online version contains supplementary material available at 10.1186/s13046-025-03462-z.

## Introduction

Lung cancer is one of the most common malignancies worldwide and has a high mortality rate [1]. Lung adenocarcinoma (LUAD) is the main histological subtype of lung cancer, and approximately 40% of Asian patients with LUAD carry activating mutations in the tyrosine kinase domain of the epidermal growth factor receptor (EGFR) [2–4]. EGFR tyrosine kinase inhibitors (TKIs), including the first-generation (gefitinib and erlotinib), second-generation (afatinib), and third-generation (osimertinib) TKIs [5, 6], have been recommened as standard first-line treatment for patients with advanced LUAD with activating EGFR mutations world wide. However, patients invitably develop acquired resistance to EGFR-TKIs, which limits the efficacy of these TKIs in treating EGFR-mutated LUAD. Osimertinib is widely used to treat patients with advanced EGFR-mutated LUAD who experience disease progression during therapy with other EGFR-TKIs [7]. Nevertheless, similar to other EGFR-TKIs, osimertinib resistance presents major obstacles to the treatment of LUAD. These factors highlight the urgent need to further explore the mechanisms underlying osimertinib resistance and identify more effective therapeutic targets than can improve the prognosis of patients with EGFR-mutated LUAD.

Long non-coding RNAs (lncRNAs) are a class of RNAs with a length of more than 200 nucleotides and no protein-coding potential. They play crucial roles in cancer biology, including metastasis and tumourigenesis, thus making them potential therapeutic targets or predictive biomarkers [8]. Accumulating evidence suggests that lncRNAs play key roles in conferring resistance to EGFR-TKIs [9]. For example, the lncRNA CASC9 promotes gefitinib resistance in non-small cell lung cancer via recruiting enhancer of zeste homolog 2 and repressing dual specificity phosphatase 1 [10]. The LncRNA BC009639 promotes lung cancer epithelial–mesenchymal transition and EGFR-TKI resistance by facilitating splicing of inositol monophosphatase domain containing 1 [11]. However, the roles and mechanisms of action of these lncRNAs in osimertinib-resistant LUAD remain unclear.

APCDD1L-AS1, an lncRNA, has been reported to induce drug resistance in LUAD and oral squamous cell carcinoma [12, 13]. In LUAD, the upregulation of APCDD1L-AS1 induces icotinib resistance by sponging with miR-1322/miR-1972/miR-324-3p [13]. APCDD1L-AS1 (encoded at 20q13.32) consists of seven exons with a full-length of 2099 nucleotides and is predominantly localised in the cytoplasm [13]. The cellular localisation of lncRNAs is central to determining their functional effects [14]. In the cytoplasm, lncRNAs can modulate gene and protein expression at the post-transcriptional level by interacting with nucleic acids or proteins [15]. Cytoplasmic lncRNAs are competitive endogenous RNAs that regulate gene expression by sponging microRNAs [16]. In addition, cytoplasmic lncRNAs can regulate cancer-related pathways by binding to key signalling proteins to influence protein–protein interactions, modulate protein stability, or localise directly within cellular compartments [8, 17]. We recently reported that the lncRNA ITPR1-AS1 activates the cRaf-MEK-ERK cascade by directly interacting with DEAD-box polypeptide 3 in the cytoplasm [17]. Previous studies focused on APCDD1L-AS1 sponging by miRNAs. However, the biological roles and mechanisms of action of APCDD1L-AS1 in osimertinib-resistant LUAD remain unclear.

The tricarboxylic acid (TCA) cycle is the central pathway that produces cellular energy and the precursors for biosynthetic pathways. Emerging evidence indicates that the TCA cycle is a critical contributor to cancer metabolism and tumorigenesis [18]. Abnormalities in metabolic enzymes are commonly detected in various cancer types, and these abnormalities generally affect the integrity of the TCA cycle to malignant progression [18, 19]. Dihydrolipoamide S-succinyltransferase (DLST) is the E2 transferase of α-ketoglutarate dehydrogenase complex (KGDHC) that mediates the irreversible conversion of α-ketoglutarate to succinyl-CoA while producing nicotinamide adenine dinucleotide (NADH) for oxidative phosphorylation (OXPHOS) [20]. Interestingly, previous studies have shown that α-ketoglutarate and succinate levels are increased in osimertinib-tolerant cells [18], and that DLST is upregulated in many cancer types and plays important roles in cancer evolution [19–21]. However, the mechanism underlying aberrant metabolic pathways in osimertinib-resistant LUAD remains largely unknown.

In this study, we aimed to explore the expression and influence of APCDD1L-AS1 in osimertinib-resistant LUAD and further investigate the downstream mechanisms that control its binding to proteins. We also investigated the underlying upstream mechanisms to gain novel insights into osimertinib-resistant LUAD.

## Materials and methods

### Patients and specimens

All tissues including paired para-tumour and LUAD tissue samples, were collected from the Nanjing Medical University Affiliated Cancer Hospital (Jiangsu Cancer Hospital, Nanjing, China) between January 2019 and December 2024. The clinical and histological features of these specimens have been previously confirmed. The use of clinical samples, with patient consent, was approved by the Institutional Research Ethics Committee. Detailed clinicopathological characteristics are listed in Supplementary Table S1&S2.

### Data source and process

Two microarray datasets (GSE163913, GSE223009) across different platforms were obtained from the Gene Expression Omnibus (GEO). Moreover, The Cancer Genome Atlas (TCGA) level 3 RNA-Seq by Expectation-Maximization (RSEM) normalized RNA-seq data and clinical phenotype data were accessed from University of California, Santa Cruz (UCSC) Xena (https://xenabrowser.net/datapages/).

The GEO and TCGA data were combined to identify significantly upregulated genes. Data from two the GEO datasets (GSE163913and GSE223009) were integrated into a single set. With a threshold of fold change > 2 and adjusted *P* < 0.001, significantly up-regulated genes were screened out using the ‘Limma’ R package. A TCGA dataset containing LUAD and normal samples with RSEM-normalised read counts was used as another set for the screening significantly upregulated genes. With a fold change threshold > 2 and false discovery rate (FDR) Q < 0.001, significantly upregulated genes were acquired using the ‘DEseq2’ R package.

### Cell lines and culture

EGFR-mutant LUAD cell lines (PC9, H1975, HCC827) were purchased from Shanghai Institutes for Biological Science (Shanghai, China). Osimertinib-resistant cell lines (PC9/OR, H1975/OR, HCC827/OR) were kindly provided by Professor Wang Xuerong (Pharmacological Laboratory, Nanjing Medical University, Nanjing, China). The osimertinib-resistant PC9/OR, H1975/OR and HCC827/OR cell lines were established by exposing PC9, H1975, and HCC827 cells to progressively increasing concentrations of osimertinib (1-1000 nmol/L) for approximately 6 months. First, the cells were exposed to 1 nmol/L osimertinib until the survival rate decreased to 30%, at which point the drug was withdrawn for recovery. When the half-maximal inhibitory concentration (IC50) increased 50-fold and 500-fold, the initial dose was increased to 5 nmol/L, and subsequently increased to 20 and 50 nmol/L, respectively. Parallel-cultured untreated cells were defined as parental cells (PC9-P, H1975-P and HCC827-P) [22]. DMEM or RPMI-1640 medium (KeyGene, Nanjing, Jiangsu, China) was used to culture the cells. All cells were cultured in medium supplemented with 10% certified heat-inactivated foetal bovine serum (FBS; Gibco, New York, NY, USA), penicillin (100 U/mL), and streptomycin (100 mg/mL) at 37 °C in a humidified 5% CO_2_ atmosphere.

### IC50 assays

Cells (5 × 10^3^) were seeded into 96-well plates with 200 µL of complete medium, after which osimertinib was added at concentrations of 0.25, 0.5, 1.0, 2.0, and 4.0 µM, in addition to an equal-volume control group, with three replicates per group. Cell proliferation was analysed using cell counting kit-8 (CCK-8) assays (Vazyme, Nanjing, Jiangsu, China) at 48 h in accordance with the manufacturer’s instructions. The absorbance of the plates was measured at 490 nm. The IC50 values were analysed using GraphPad Prism 8.3 software.

### Cell proliferation, migration, and invasion assays

Cell viability was detected by adding 5% CCK8 and incubation at 37 °C for 2 h at 0, 24, 48, 72, and 96 h. The absorbance of each well was measured at 490 nm. All experiments were performed at least in triplicate. 5-Ethynyl-2′-deoxyuridine (EdU RiboBio, Guangzhou, Guangdong, China), colony-formation, Transwell, Matrigel, and wound-healing assays were performed as previously described [17].

### Tumour subcutaneous xenografts

Sixteen BALB/c nude mice aged 4–6 weeks were purchased and divided into two groups that received PC9-P or PC9/OR cells through subcutaneous injection at a dose of 3 × 10^6^ cells/150 µL. When the average tumour volume reached 60 mm^3^, each group of mice was further randomly divided into two groups and subjected to intragastric administration of either normal saline or osimertinib (20 mg/kg/day, Cat#HY-15772; MedChemExpress) by gavage every 2 days.

### Small interfering rnas, plasmid construction and cell transfection

Small interfering RNAs (siRNAs) were obtained from RiboBio (Guangzhou, China). Plasmid vectors (pcDNA3.1-APCDD1L-AS1, pcDNA3.1-DLST, and the empty pcDNA3.1 vector) were obtained from Realgene Biotechnology (Nanjing, China). Transfection of siRNAs and plasmids was performed using a Lipofectamine™ 3000 kit (Invitrogen, Carlsbad, CA, USA) in accordance with the manufacturer’s protocol. All siRNA sequences are listed in Supplementary Table S3.

### Lentiviral transfection

Lentiviral (LV)- hort hairpin negative control (shNC) and LV-sh APCDD1L-AS1 were used to transfect PC9/OR cells. The short hairpin RNA sequences used to silence the corresponding genes were the same as siAPCDD1L-AS1 3#. After transduction with the lentiviral particles, PC9/OR cells were cultured in RPMI-1640 supplemented with 10% FBS. Inducible cell sublines were established by puromycin selection.

### Cell cycle assay and apoptosis assay

For the cell cycle assay, cells were trypsinised into a single-cell suspension, rinsed with ice-cold phosphate-buffered saline (PBS), and fixed in ice-cold 70% ethanol overnight. The cells were then treated with a Cell Cycle Assay Kit (KeyGene, Nanjing, Jiangsu, China) in accordance with the manufacturer’s instructions. Cell apoptosis was assessed using the Annexin V-APC/PI Apoptosis Detection Kit (KeyGene, Nanjing, Jiangsu, China). A flow cytometer (FACScan; BD Biosciences, Mountain View, CA, USA) equipped with CellQuest software was used to analyse cell cycle and apoptosis.

### Western blotting, immunofluorescence, chromogenic in situ hybridization and immunohistochemistry assays

Western blotting, immunofluorescence, chromogenic in situ hybridisation (CISH), and immunohistochemistry (IHC) assays were performed as described previously [17]. All the antibodies used are listed in Supplementary Table S4.

### RNA extraction and quantitative reverse transcription-polymerase chain reaction

RNA extraction and quantitative reverse transcription (qRT)-polymerase chain reaction (PCR) were performed as previously described [17]. The PCR primer sequences are listed in Supplementary Table S5.

### Nuclear and cytoplasmic RNA separation and fluorescence in situ hybridisation assays

Nuclear and cytoplasmic RNA separation and fluorescence in situ hybridisation (FISH) assays were performed as previously described [17].

### RNA pull-down assays and mass spectrometry analyses

RNA pull-down assays were conducted using a Pierce™ Magnetic RNA-Protein Pull-Down kit (CAT. No. 20164; Thermo Fisher Scientific, Waltham, MA, USA) in accordance with the manufacturer’s instructions, as described previously [17]. In brief, biotinylated RNAs were mixed with dynabeads and incubated at 4℃ overnight, followed by incubation with cell lysates for 1 h at 4℃. The bead–RNA–protein complexes were diluted with sodium dodecyl sulphate (SDS) buffer. Finally, the retrieved proteins were analysed by gradient gel electrophoresis followed by mass spectrometry.

### RNA Immunoprecipitation assay

RNA immunoprecipitation (RIP) assays were conducted using the EZMagna RIP™ Kit (No.17–700, Merck, Darmstadt, Hessian, Germany) in accordance with the manufacturer’s instructions. The retrieved RNAs were subjected to qRT-PCR analysis using primers specific for APCDD1L-AS1. IgG (Abcam) controls were assayed simultaneously to confirm that the RNA specifically interacted with DLST.

### Chromatin Immunoprecipitation

The chromatin immunoprecipitation (ChIP) assay was performed using a ChIP kit (CAT.NO.17-10086, Merck, Darmstadt, Hessian, Germany) in accordance with the manufacturer’s protocol. First, after crosslinking with formaldehyde, PC9/OR and H1975/OR cells (1 × 10^7^) were sonicated to produce chromatin fragments of 200–400 bp. Supernatants were immunoprecipitated with hypoxia-inducible factor (HIF)-1α (10 µg). IgG (Millipore, Billerica, MA, USA, 2 µg) was used as the control. After elution and purification, DNA was analysed using RT-qPCR. The primers used for ChIP-qPCR are listed in Supplementary Table S5.

### Citrate, oxaloacetate, succinate, α-ketoglutarate and succinyl-CoA measurement

For measuring citate, oxaloacetate and succinate production, cells (2 × 10^6^) were rapidly homogenized with 100 mL of assay buffer. Then, measure these TCA cycle intermediates concentration by using Citrate Assay Kit (Cat NO. MAK057, Merck, Darmstadt, Hessian, German); Oxaloacetate Assay Kit (Cat NO. MAK070, Merck, Darmstadt, Hessian, German); Succinate Assay Kit (Cat NO. MAK335, Merck, Darmstadt, Hessian, German); α-ketoglutarate Kit (Cat NO. BC4704, Solarbio, Beijing, China) and Succinyl-CoA Kit (Cat NO. MM-51024h1, MEIMIAN, Jiangsu, China). The samples were subjected to set up three intricate wells. Plates were read at 570 nm absorbance. The measured data were processed with the GraphPad Prism 8.3 software.

### 769–815 Nt of APCDD1L-AS1 knock out cell lines

To knock out (KO) 769–815 nt of APCDD1L-AS1 in osimertinib-resistant LUAD cells, six single-guide RNAs (sgRNAs) were designed (Supplementary Fig. S1A), the sgRNA sequences are listed in Supplementary Table S6. After infection and testing, sgRNA1 (KO1) and sgRNA5 (KO5) were selected to construct expression plasmids and packaging LVs to knockout the specified region of the APCDD1L-AS1 gene. The PC9/OR and H1975/OR cell lines were infected with lenti CRISPR v2 LV and selected with puromycin (1 µg/mL). After selection, the cells were maintained with puromycin (1 µg/mL). Isolated single clones were subjected to PCR and DNA sequencing for knockout validation. Sanger sequencing confirmed the deletion of 769–815 nt of APCDD1L-AS1 in the knockout single-clone cells, which was the result of the combined action of the KO1 and KO5 knockout sites (Supplementary Fig. S1B). Sanger sequencing data are provided in Supplementary Material (0013_32025052401683_(APCDD1L-KO15-13)_[APCDD1L-DT-PF1].ab1). The APCDD1L-AS1 (769–815 nt) KO cell line was used for subsequent experiments. The negative control cell line was generated by infection of PC9/OR and H1975/OR cells with a control LV containing a random sequence that did not target any genes (vector).

### Oxygen consumption rate assay

Briefly, cells were seeded in 96-well Seahorse assay plates at a concentration of 1.0 × 10^5^ cells/well and cultured overnight for attachment. The oxygen consumption rate (OCR) was measured using a Seahorse XFe96 Analyzer (Agilent, California, CA, USA) with a Seahorse XF Cell Mito Stress Test Kit (Agilent, cat#: 103015-100), in accordance with the manufacturer’s instructions.

### Luciferase reporter assay

The Pierce™ Gaussia Luciferase Flash Assay Kit-100Rxns (CAT. no. 16158; Thermo Fisher Scientific, Waltham, MA, USA) was used in accordance with the manufacturer’s protocol. Cells (10,000) were seeded into 96-well plates. The plates were incubated overnight at 37 °C and 5% CO_2_. PC9/OR and H1975/OR cells were transfected with Gaussia luciferase plasmid. The cells were incubated in a cell culture incubator at 37 °C and 5% CO_2_ for 48 h. The transfected cells were removed from the medium within 48 h of transfection. The cells were then rinsed with 100 µL/well of 1X DPBS buffer, after which the DPBS was aspirated and 100 µL/well of 1X cell lysis buffer was added. The plate was spun at a medium speed on the shaker platform for 15 min. Next, 20 µL/well of cell lysate or medium was added to a black, opaque 96-well plate. After programming the photometer, 50 µL of working solution was added to each well. Light output was detected as soon as the reagent was added.

### Hypoxia experiment

First, 1 × 10^6^ cells were seeded in a flask and incubated overnight in a standard incubator at 37 °C and 5% CO₂ until the cells were completely adherent. The flask was placed in an anoxic incubator containing 1% O₂ and 5% CO₂ for 37 °C for the following time points: 0 h (no hypoxic exposure, control); 4 h; 8 h; 16 h; 24 h; 48 h. At each time point (0, 4, 8, 16, 24, and 48 h), the cells were collected for protein and RNA extraction. Validation was performed using western blotting and qRT-PCR.

### Statistical analysis

Statistical analyses were performed using SPSS version 22.0 (SPSS), GraphPad Prism 8.0 (GraphPad Software Inc., San Diego, CA, USA), Stata 12 (StataCorp LLC, College Station, TA, USA), and R software (version 4.1.2; http://www.r-project.org). Student’s t-test was used for analysis of continuous variables, and the χ^2^ test or Fisher’s exact test was performed to analyse categorical variables. Kaplan–Meier plots and log–rank tests were used for survival analyses. Univariate and multivariate Cox proportional-hazard regression models were used to analyse independent prognostic factors. The in vitro data are presented as mean ± standard deviation for three independent experiments. The significance levels for differences were set at *P < 0.05, **P < 0.01, and ***P < 0.001.

## Results

### APCDD1L-AS1 is significantly up-regulated in osimertinib-resistant LUAD

**Fig. 1:**
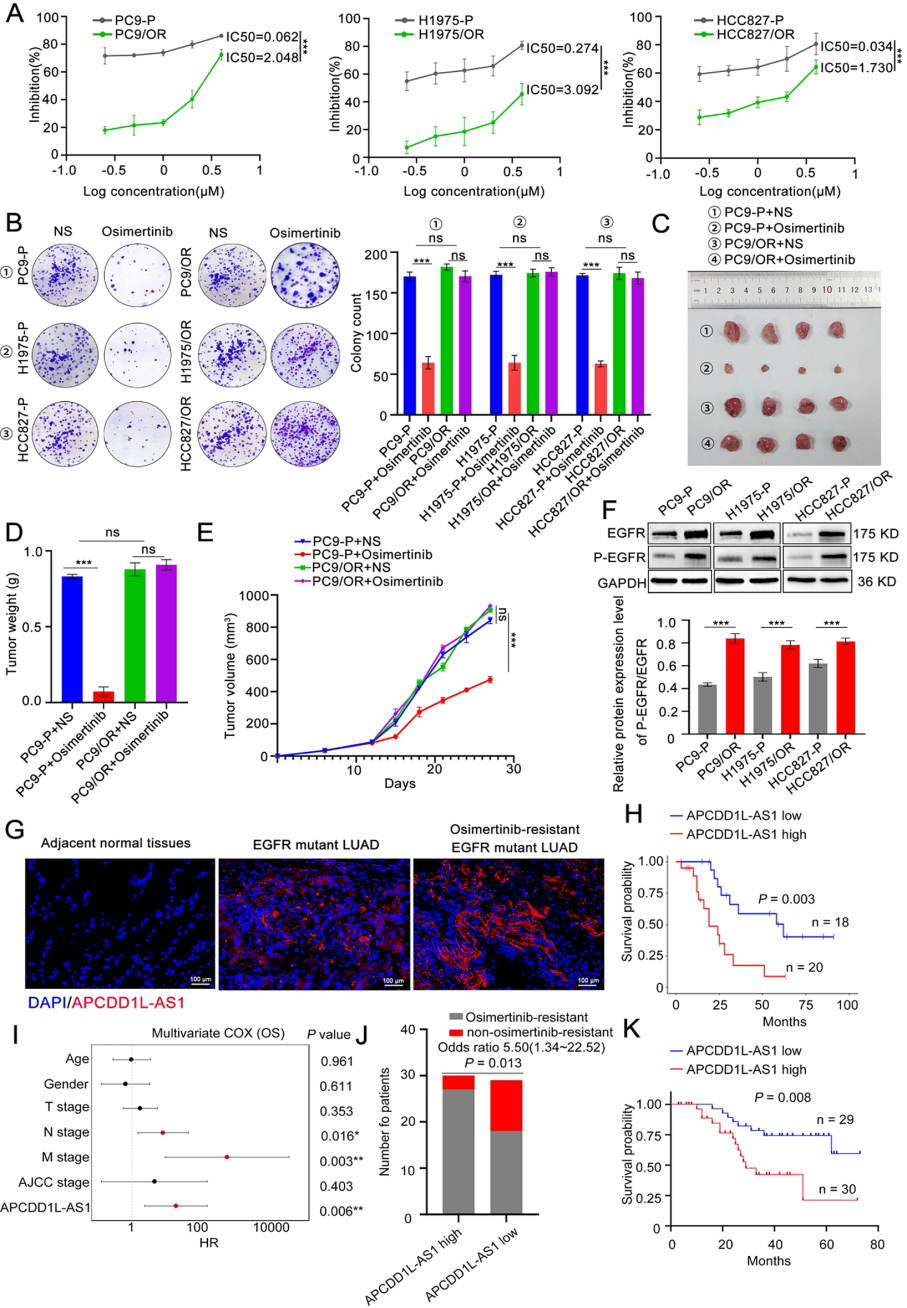
APCDD1L-AS1 is significantly up-regulated in osimertinib-resistant lung adenocarcinoma (LUAD). (**A**) The osimertinib sensitivity in osimertinib-resistant LUAD cells and their parent cells treated with different osimertinib doses for 48 h was assessed using the CCK-8 test. (**B**) The colony formation assay was used to examine the proliferation ability of osimertinib-resistant LUAD cells and their parent cells under 2µM osimertinib or normal saline (NS). (**C-E**) Tumor volume, weight, and growth curves of the subcutaneous tumor mouse models of osimertinib-resistant cells and their parental cells treated with or without osimertinib (*n* = 4). (**F**) The protein level of EGFR and p-EGFR in the osimertinib-resistant LUAD cells and their parental cells was detected. (**G**) RNA chromogenic in situ hybridization (CISH) was used to analyze the expression of APCDD1L-AS1 in adjacent normal tissues, EGFR mutant LUAD and osimertinib-resistant LUAD tissue samples. (**H**) Kaplan-Meier analysis indicates that osimertinib-resistant LUAD patients with higher levels of APCDD1L-AS1 expression exhibited worse overall survival (OS). (**I**) Multivariate Cox regression analysis revealed that OS was independently at risk from APCDD1L-AS1. (**J**) Analysis of the association between APCDD1L-AS1 expression in the patients with and without resistance to osimertinib. (**K**) Kaplan-Meier analysis demonstrates that EGFR-mutant LUAD before osimertinib treatment patients with higher levels of APCDD1L-AS1 expression exhibited worse OS. Data presented as mean ± standard error of mean (*n* = 3; statistical analysis by GraphPad Prism 8.0 (GraphPad Software Inc., San Diego, CA, USA); * *P* < 0.05; ** *P* < 0.01; *** *P* < 0.001)

First, we evaluated the sensitivity of the parental cells (PC9-P, H1975-P, and HCC827-P) and osimertinib-resistant cells (PC9/OR, H1975/OR, and HCC827/OR) to osimertinib. The CCK8 assays suggested that the sensitivity of osimertinib-resistant cells to osimertinib was significantly lower than that of parent cells in a dose and time dependent manner (Fig. 1A). As shown in Fig. 1B the colonies of the parent cells were significantly smaller than those of the osimertinib-resistant cells after osimertinib treatment. The size and the weight of the subcutaneous tumour grafts in the PC9-P cell group were smaller than those in the PC9/OR cell group after osimertinib treatment (Fig. 1C-E). Furthermore, western blot assays showed that the expression levels of EGFR and phospho-EGFR in osimertinib-resistant cells were higher than those in parental cells (Fig. 1F). These results indicate that the osimertinib-resistant phenotype in LUAD cells is credible.

To determine the contribution of lncRNAs to osimertinib resistance in LUAD, we performed an integrated analysis of lncRNAs in LUAD/adjacent normal tissues from TCGA and PC9-P&PC9/OR and HCC827-P&HCC827/OR cells of GSE163913 and GSE223009 from the GEO datasets (Supplementary Fig. S2A). A total of five candidate upregulated genes were identified as robust significantly elevated genes in osimertinib-resistant LUAD after overlapping 273 significantly upregulated genes in the GEO datasets and 3546 remarkably elevated genes in the TCGA datasets. Among them, APCDD1L-AS1 attracted our attention given its significant differential expression, and the fact that its high expression predicted worse survival (Supplementary Fig. S2B-D). In addition, the expression of APCDD1L-AS1 was also analysed using RNA CISH in 48 pairs of LUAD/ adjacent normal tissues and 38 osimertinib-resistant LUAD tissue samples. The results showed that APCDD1L-AS1 expression was significantly upregulated in osimertinib-resistant LUAD tissues with EGFR mutations in comparison with adjacent normal tissues **(**Fig. 1G**)**. Additionally, Kaplan–Meier analysis showed that patients with osimertinib-resistant LUAD with high APCDD1L-AS1 expression (as determined by a cutoff score of the median) had worse overall survival (OS) than those with low APCDD1L-AS1 expression (*P* = 0.003; Fig. 1H). Multivariate Cox regression analysis revealed that high APCDD1L-AS1 expression served as an independent risk factor for OS in osimertinib-resistant LUAD (hazard ratio [HR] = 19.1, 95% confidence interval [CI] = 2.33–155., *P* = 0.006; Fig. 1I). The expression of APCDD1L-AS1 was also analysed using RNA CISH in tissue specimens obtained from 59 patients with EGFR-mutant LUAD before osimertinib treatment (Supplementary Fig. S2E). An independent blinded pathological examination revealed a statistically significant association between APCDD1L-AS1 expression and a decrease in the objective response rate (Fig. 1J). Furthermore, high expression of APCDD1L-AS1 (as determined by a cutoff score of the median) in cancer cells from pre-treatment samples was significantly correlated with poorer progression-free survival following osimertinib treatment (Fig. 1K), indicating that not only is APCDD1L-AS1 expression induced by treatment, but its high baseline expression prior to treatment can also occur independently of osimertinib administration, potentially leading to resistance. In LUAD cell lines, APCDD1L-AS1 expression was higher in osimertinib-resistant cells (PC9/OR, H1975/OR and HCC827/OR) than in osimertinib-sensitive cell lines (PC9-P, H1975-P and HCC827-P) (Supplementary Fig. S2F). These results indicated that APCDD1L-AS1 was frequently upregulated in osimertinib-resistant LUAD cells and tissues.

### APCDD1L-AS1 promotes osimertinib resistance of LUAD cells

**Fig. 2:**
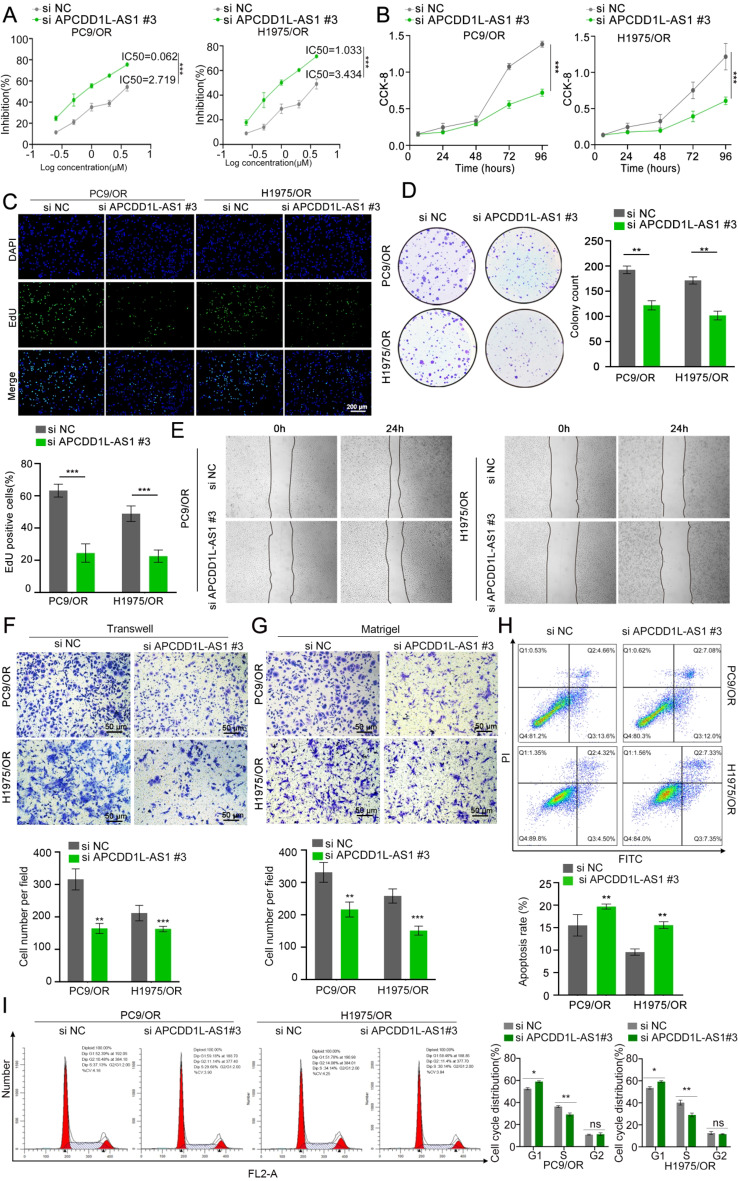
APCDD1L-AS1 promotes osimertinib resistant of LUAD cells. (**A**) The osimertinib sensitivity in PC9/OR and H1975/OR cells transfected with APCDD1L-AS1 siRNA or control for 48 h was assessed using the CCK-8 test. (**B-C**) CCK8 and EdU assays showed that APCDD1L-AS1 silencing significantly reduced the proliferation of PC9/OR and H1975/OR cells. (**D**) APCDD1L-AS1 silencing attenuated the colony formation capacity of PC9/OR and H1975/OR cells. (**E-G**) APCDD1L-AS1 knockdown dramatically reduced the migration and invasive abilities of PC9/OR and H1975/OR cells, according to wound healing, Transwell, and Matrigel^®^ assays. (**H-I**) According to flow cytometry tests, APCDD1L-AS1 silencing decreased apoptosis and caused G1 phase cell cycle arrest in PC9/OR and H1975/OR cells. Data presented as mean ± standard error of mean (*n* = 3; statistical analysis by GraphPad Prism 8.0 (GraphPad Software Inc., San Diego, CA, USA); * *P* < 0.05; ** *P* < 0.01; *** *P* < 0.001)

To explore the biological functions of APCDD1L-AS1 in the osimertinib-resistant LUAD cells, we designed three siRNAs and transfected them into osimertinib-resistant LUAD cells to knockdown the expression of APCDD1L-AS1. Si APCDD1L-AS1 #3 was used in subsequent assays because it exhibited better efficacy in both PC9/OR and H1975/OR cells (Supplementary Fig. S2G). As shown in Fig. 2A, APCDD1L-AS1 silencing significantly enhanced osimertinib sensitivity. CCK8, EdU, and colony-formation assays showed that silencing APCDD1L-AS1 greatly inhibited the proliferation of PC9/OR and H1975/OR cells (Fig. 2B-D). Wound-healing, Transwell, and Matrigel^®^ assays indicated that knockdown of APCDD1L-AS1 significantly attenuated the migration and invasive abilities of osimertinib-resistant LUAD cells (Fig. 2E-G). In addition, flow cytometry analysis showed that knockdown of APCDD1L-AS1 reduced apoptosis and led to G1 phase cell cycle arrest in PC9/OR and H1975/OR cells (Fig. 2H-I). Conversely, exogenous overexpression of APCDD1L-AS1 induced the resistance of PC9/OR and H1975/OR cells to osimertinib (Supplementary Fig. S 3 A). Furthermore, overexpression of exogenous APCDD1L-AS1 in osimertinib-resistant LUAD cells promoted cell proliferation, colony formation, migration, invasiveness, and cell cycle progression while reducing apoptosis (Supplementary Fig. S3 B-I). Additionally, the CCK8, EdU, colony-formation, wound-healing, Transwell, and Matrigel^®^ assays showed that silencing APCDD1L-AS1 attenuated the proliferation, migration, and invasiveness of PC9-P and H1975-P cells, while exogenous APCDD1L-AS1 overexpression promoted these characteristics (Supplementary Fig. S4&S5). In vitro experiments confirmed that APCDD1L-AS1 enhances osimertinib resistance in LUAD cells.

### APCDD1L-AS1 directly interacted with DLST

**Fig. 3:**
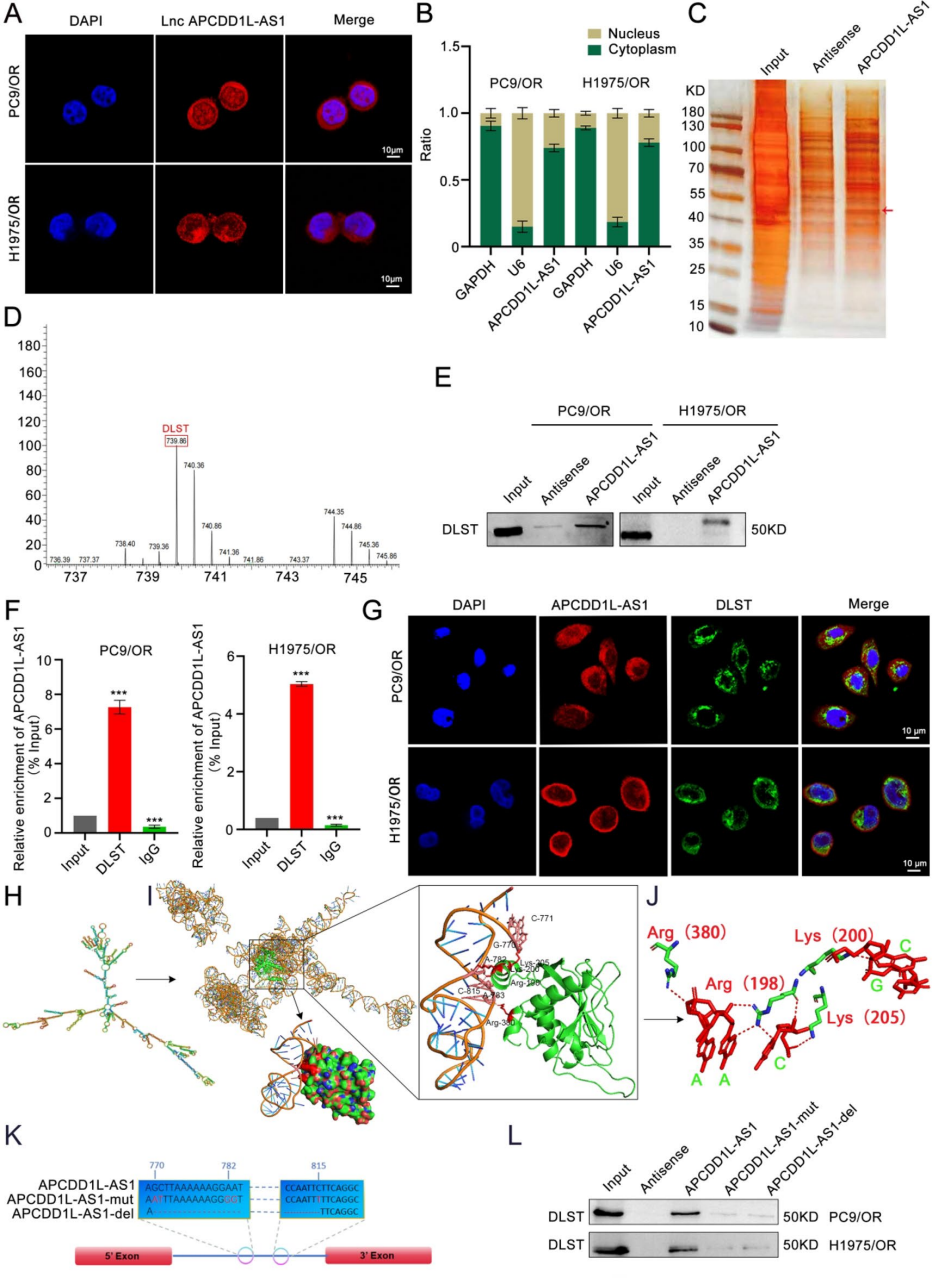
APCDD1L-AS1 directly interacted with DLST. (**A-B**) APCDD1L-AS1 in PC9/OR and H1975/OR cells using RNA fluorescence in situ hybridization (FISH) and Nuclear and cytoplasmic RNA separation analysis, Scale bar = 10 μm. (**C-D**) Assays for biotinylated APCDD1L-AS1 related proteins using RNA pulldown and mass spectrometry assays. (**E-F**) RNA pulldown and RIP analysis of the interaction between APCDD1L-AS1 and DLST. (**G**) FISH and immunofluorescence staining assays showed the intracellular localization of APCDD1L-AS1 and DLST. (**H**) Secondary structures of APCDD1L-AS1 were determined by the minimum free energy algorithm implemented in RNAfold. (**I-J**) Graphical representation of three-dimensional structures of APCDD1L-AS1 and DLST docking models with a zoom-in image of the binding interface. (**K**) Two APCDD1L-AS1 vectors constructed in which the predicted binding sites were mutated (APCDD1L-AS1-mut) or depleted (APCDD1L-AS1-del). (**L**) RNA-pull down assays revealed that DLST was physically bind to wild type APCDD1L-AS1 but not APCDD1L-AS1-mut or APCDD1L-AS1-del. Data presented as mean ± standard error of mean (*n* = 3; statistical analysis by GraphPad Prism 8.0 (GraphPad Software Inc., San Diego, CA, USA); * *P* < 0.05; ** *P* < 0.01; *** *P* < 0.001)

To investigate the cellular localisation of APCDD1L-AS1, we performed RNA FISH and subcellular fractionation assays. As shown in Fig. 3A-B, APCDD1L-AS1 was mainly expressed in the cytoplasm of PC9/OR and H1975/OR cells, suggesting that APCDD1L-AS1 may perform its biological function at the post-transcriptional level. Next, RNA pull-down and mass spectrometry assays indicated that DLST may be the main binding protein for APCDD1L-AS1 (Fig. 3C-D, Supplementary Table S7). Furthermore, RNA pull-down, RIP, FISH, and immunofluorescence staining assays revealed that APCDD1L-AS1 formed a complex with DLST and co-localised in the cytoplasm of PC9/OR and H1975/OR cells (Fig. 3E-G). The minimum free energy algorithm implemented in RNAfold was used to determine the secondary structures of APCDD1L-AS1 (Fig. 3H). After submitting the secondary structure of APCDD1L-AS1 to a 3DRNA web server, the 3D structure of APCDD1L-AS1 was generated. Next, in silico molecular docking between APCDD1L-AS1 and DLST was performed using the HDOCK web server. As shown in Fig. 3I, DLST could bind to the double-helix of the hairpin structure formed by APCDD1L-AS1. PyMOL visualisation showed that hydrogen bonds may form between Lys^200^ of DLST and GC^770–771^ of APCDD1L-AS1, Arg^198^ of DLST and AA^782–783^ of APCDD1L-AS1, and Lys^205^ of DLST and C^815^ of APCDD1L-AS1, suggesting that the 769–815 nt region of APCDD1L-AS1 interacts with the 198–205 aa segment of DLST (Fig. 3I-K). Next, we constructed two APCDD1L-AS1 vectors in which the predicted binding sites were either mutated (APCDD1L-AS1-mut) or depleted (APCDD1L-AS1-del) to validate these predicted binding sites. RNA pull-down assays showed that DLST physically binds to wild-type APCDD1L-AS, but not APCDD1L-AS1-mut or APCDD1L-AS1-del (Fig. 3L), which suggested that 769–815 nt was the region in APCDD1L-AS1 that interacted with DLST. Our data indicate that 769–815 nt of APCDD1L-AS1 interacted with DLST.

### APCDD1L-AS1 drives the TCA cycle by inhibiting ubiquitination and degradation of DLST protein

**Fig. 4:**
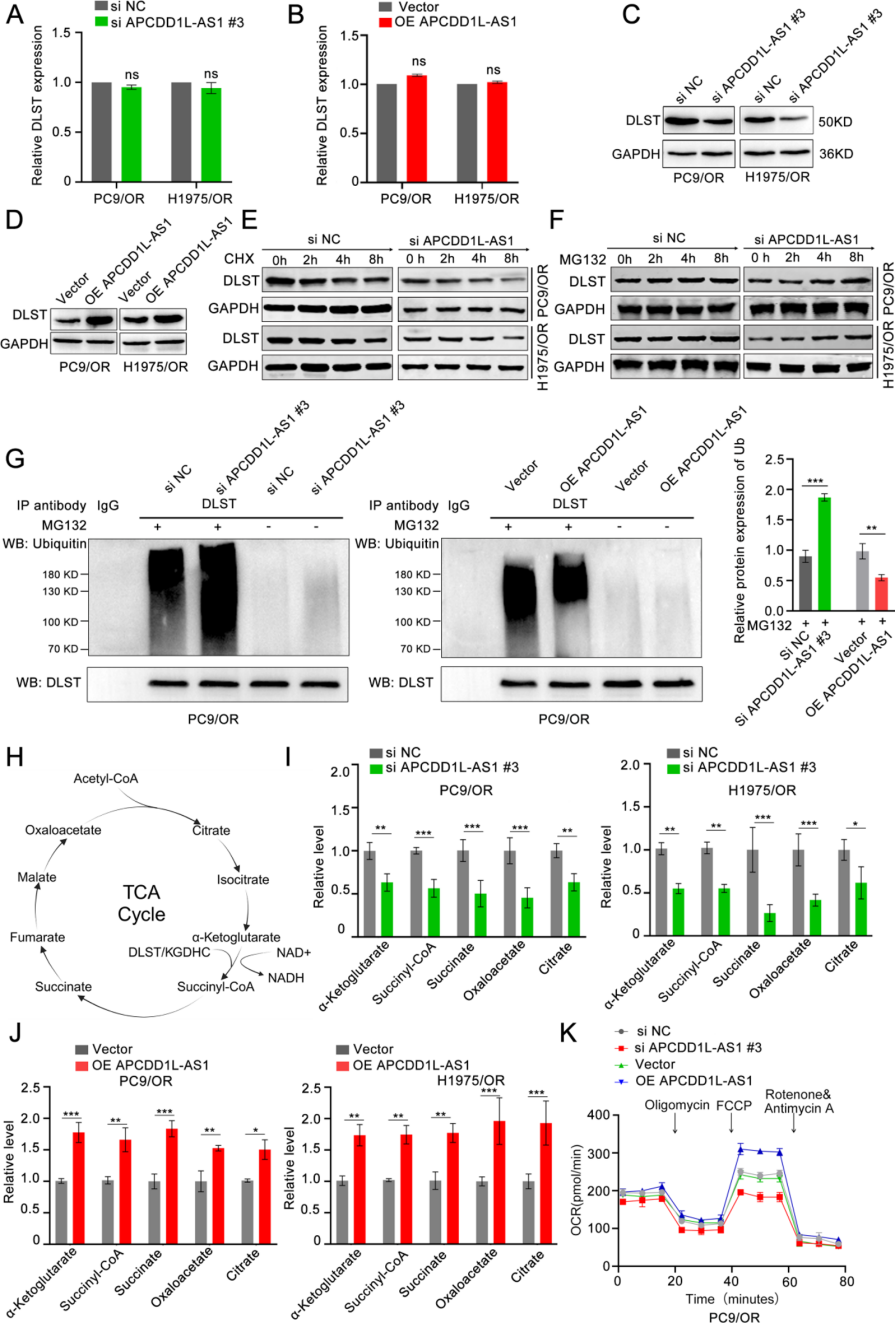
APCDD1L-AS1 drives tricarboxylic acid cycle by inhibiting ubiquitination and degradation of DLST protein. (**A-B**) qPCR assay showed DLST mRNA levels were not significantly affected by APCDD1L-AS1 knockdown or overexpression. (**C-D**) Western blot assay verified the knockdown or overexpression of APCDD1L-AS1 significantly affected DLST protein levels. (**E-F**) PC9/OR and H1975/OR cells transfected with APCDD1L-AS1 siRNA or control. After transfection for 48 h, several cells were treated with cycloheximide (CHX; 10 µg/mL) or MG132 (10 µM) for 0, 2, 4, 8 h, and western blot analysis was then used to examine DLST expression. (**G**) PC9/OR cells were transfected with control, APCDD1L-AS1 siRNA or OE-APCDD1L-AS1. After transfection, cells were treated with either DMSO (control) or MG132 (10 µM, 16 h). Endogenous ubiquitination assays were then used to detect the ubiquitination (Ub) levels of endogenous DLST. (**H**) Schematic representation of the TCA cycle and the intermediate metabolites affected by DLST. (**I-J**) PC9/OR cells were transfected with control, APCDD1L-AS1 siRNA, or OE-APCDD1L-AS1 constructs. After transfection for 48 h, the intracellular levels of α-ketoglutarate, succinyl-CoA, succinate, oxaloacetate, and citrate were quantitatively analyzed. (**K**) Oxygen consumption rates (OCR) assays demonstrated that APCDD1L-AS1 accelerated oxidative phosphorylation (OXPHOS) in PC9/OR cells 48 h post-transfection. Data presented as mean ± standard error of mean (*n* = 3; statistical analysis by GraphPad Prism 8.0 (GraphPad Software Inc., San Diego, CA, USA); * *P* < 0.05; ** *P* < 0.01; *** *P* < 0.001)

As shown in Fig. 4A-D, APCDD1L-AS1 expression affected DLST protein levels but not mRNA levels. Phosphosite web predicted that 203 aa of DLST may be modified by ubiquitination, and it is in the region of potential binding sites for APCDD1L-AS1 and DLST. Therefore, we hypothesised that APCDD1L-AS1 covers the ubiquitination sites of DLST to protect it from ubiquitination and proteasomal degradation. To elucidate the mechanism by which APCDD1L-AS1 influences DLST protein levels, we treated PC9/OR and H1975/OR cells with the protein synthesis inhibitor cycloheximide (CHX). The results suggested that DLST was rapidly degraded in the APCDD1L-AS1-knockdown group, with protein stability also decreasing over time, suggesting that APCDD1L-AS1 prevents the degradation of DLST (Fig. 4E). As shown in Fig. 4F, treatment with the proteasome inhibitor MG132 increased the protein level of DLST and markedly rescued the decrease in DLST protein level in osimertinib-resistant LUAD cells with silenced APCDD1L-AS1, suggesting that silencing of APCDD1L-AS1 promotes the ubiquitination and degradation of DLST. Subsequently, the results of endogenous ubiquitination assays indicated that ubiquitination of DLST was greatly elevated in APCDD1L-AS1-knockdown osimertinib-resistant LUAD cells. In contrast, overexpression of exogenous APCDD1L-AS1 in PC9/OR and H1975/OR cells led to reduced ubiquitination of DLST (Fig. 4G & Supplementary Fig. S6 A). Since HSPA8 and RACK1 were identified by mass spectrometry in the APCDD1L-AS1 pull-down samples, and given their potential role in regulating the autophagic-lysosomal degradation of proteins, we investigated the effects of HSPA8 and RACK1 on DLST [22–24]. Western blot analyses confirmed that silencing of HSPA8 and RACK1 did not affect DLST protein expression (Supplementary Fig. S6 B-F). Silencing APCDDD1L-AS1 downregulated HSPA8 expression but did not affect the autophagy-lysosomal degradation of DLST (Supplementary Fig. S6 G-H). These data indicate that APCDD1L-AS1 covers the ubiquitination sites of DLST to prevent protein degradation.

Since DLST is a TCA cycle transferase, we evaluated the cellular levels of α-ketoglutarate, succinyl-CoA, succinate, oxaloacetate, and citrate during APCDD1L-AS1 knockdown or exogenous overexpression to assess the TCA cycle capacity of osimertinib-resistant LUAD cells (Fig. 4H). As shown in Fig. 4I and J, silencing of APCDD1L-AS1 greatly inhibited the TCA cycle capacity of PC9/OR and H1975/OR cells, whereas exogenous APCDD1L-AS1 overexpression in osimertinib-resistant LUAD cells promoted TCA cycle capacity. Similarly, the results of OCR assays demonstrated that APCDD1L-AS1 accelerated OXPHOS in PC9/OR and H1975/OR cells (Fig. 4K & Supplementary Fig. S6 I). These data confirm that APCDD1L-AS1 accelerates the TCA cycle and OXPHOS by binding to DLST and protecting it from ubiquitination and degradation.

Next, we generated APCDD1L-AS1 (769–815 nt)-KO in the PC9/OR and H975/OR cell lines to determine whether the 769–815 nt region of APCDD1L-AS1 is critical for DLST stabilisation. Western blot and endogenous ubiquitination assays indicated that endogenous DLST protein expression was reduced, the half-life of DLST was shortened, and ubiquitination of DLST was increased in the APCDD1L-AS1 (769–815 nt) KO cell lines (Supplementary Fig. S7 A–E). As shown in Supplementary Fig. S7 F-G, the TCA cycle capacity and OXPHOS of the APCDD1L-AS1 (769–815 nt)-KO cell lines were reduced. Furthermore, in APCDD1L-AS1 (769–815 nt)-KO osimertinib-resistant LUAD cells, APCDD1L-AS1 overexpression partially rescued the downregulation of DLST and the TCA cycle and OXPHOS capacities, whereas overexpression of APCDD1L-AS1-mut failed to reverse this effect (Supplementary Fig. S7 A-G). This indicated that the 769–815 nt region of APCDD1L-AS1 is crucial for DLST stabilisation in osimertinib-resistant LUAD cells.

### APCDD1L-AS1 contributes to acquired resistance to osimertinib and is partly dependent on regulating DLST

**Fig. 5:**
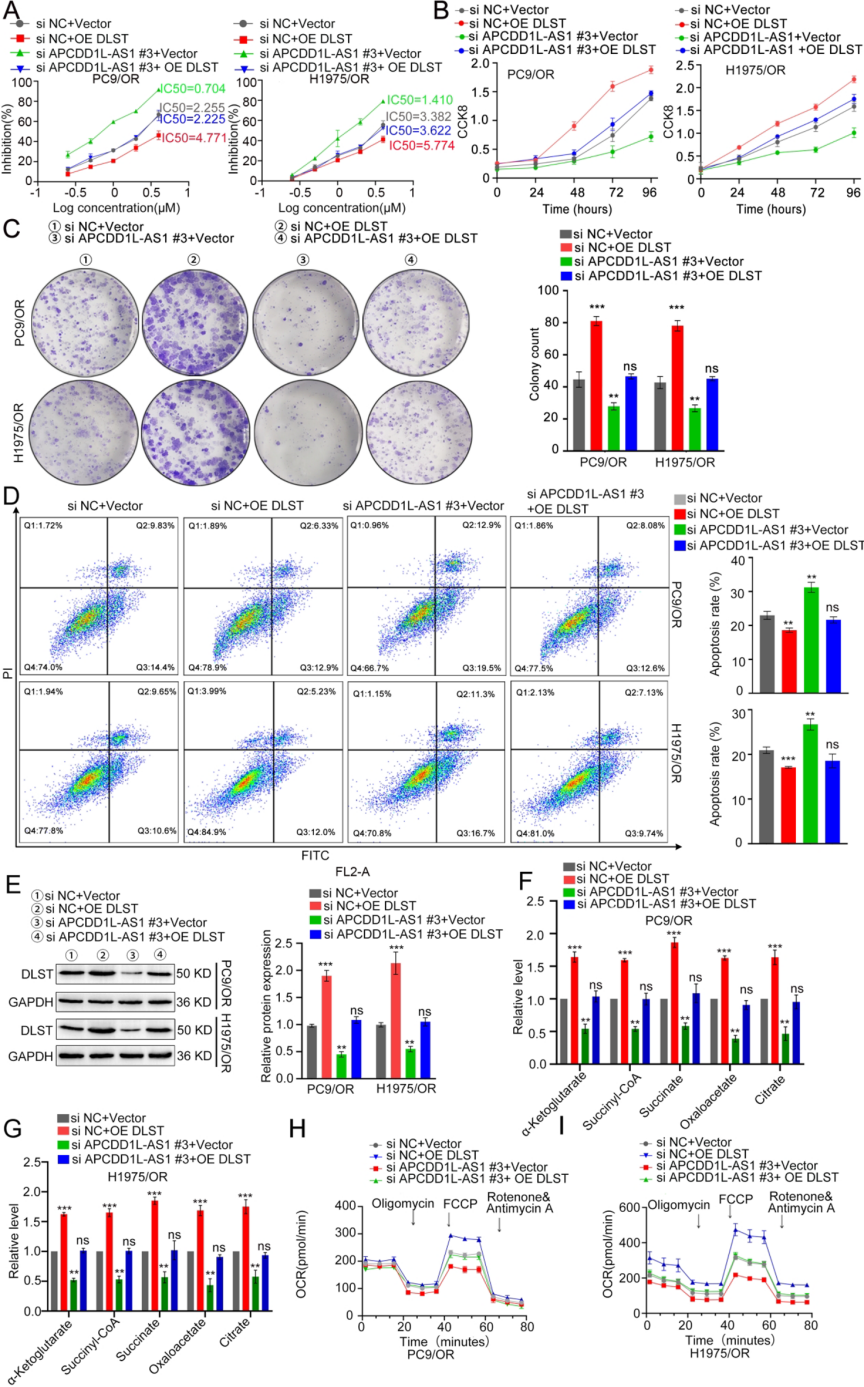
APCDD1L-AS1 contributes to acquired resistance to osimertinib and is partly dependent on regulating DLST. (**A**) CCK-8 test demonstrated that DLST restored the reduced sensitivity to osimertinib induced by APCDD1L-AS1 silencing. (**B**) According to CCK-8 experiment, DLST overexpression might reverse the increase in cell proliferation brought on by APCDD1L-AS1 silencing. (**C**) Colony formation assays showed that overexpression of DLST relieved the cell proliferation abilities induced by APCDD1L-AS1 silencing. (**D**) Flow cytometry analysis revealed that overexpression of DLST relieved the apoptosis abilities caused by APCDD1L-AS1 knockdown. (**E**) Western blot assay corroborated that DLST overexpression partially rescued the downregulation of DLST. (**F-G**) α-ketoglutarate, succinyl-CoA, succinate, oxaloacetate and citrate assays corroborated that DLST overexpression partially rescued TCA cycle. (**H-I**) OCR assays corroborated that DLST overexpression partially rescued OXPHOS ability. Data presented as mean ± standard error of mean (*n* = 3; statistical analysis by GraphPad Prism 8.0 (GraphPad Software Inc., San Diego, CA, USA); * *P* < 0.05; ** *P* < 0.01; *** *P* < 0.001)

To determine whether APCDD1L-AS1 promotes osimertinib resistance in LUAD through the APCDD1L-AS1/DLST axis, CCK8, colony-formation, EdU, Transwell-migration, Matrigel^®^ invasion, and flow cytometry assays were performed. As shown in Fig. 5A, DLST overexpression partially reversed the APCDD1L-AS1 knockdown-induced decrease in the IC50 of osimertinib in PC9/OR and H1975/OR cells. In addition, overexpression of DLST relieved cell proliferation, migration, invasiveness, and apoptosis induced by APCDD1L-AS1 silencing (Fig. 5B-D& Supplementary Fig. S8 A-C). Moreover, the results of western blot, α-ketoglutarate, succinyl-CoA, succinate, oxaloacetate, citrate, and OCR assays corroborated that DLST overexpression partially rescued the downregulation of DLST and the TCA cycle and OXPHOS capacities following APCDD1L-AS1 knockdown in osimertinib-resistant LUAD cells (Fig. 5E-I**)**. Collectively, these data indicate that DLST is a functional downstream mediator of APCDD1L-AS1.

### APCDD1L-AS1 is transcriptionally induced by HIF-1α under hypoxia

**Fig. 6:**
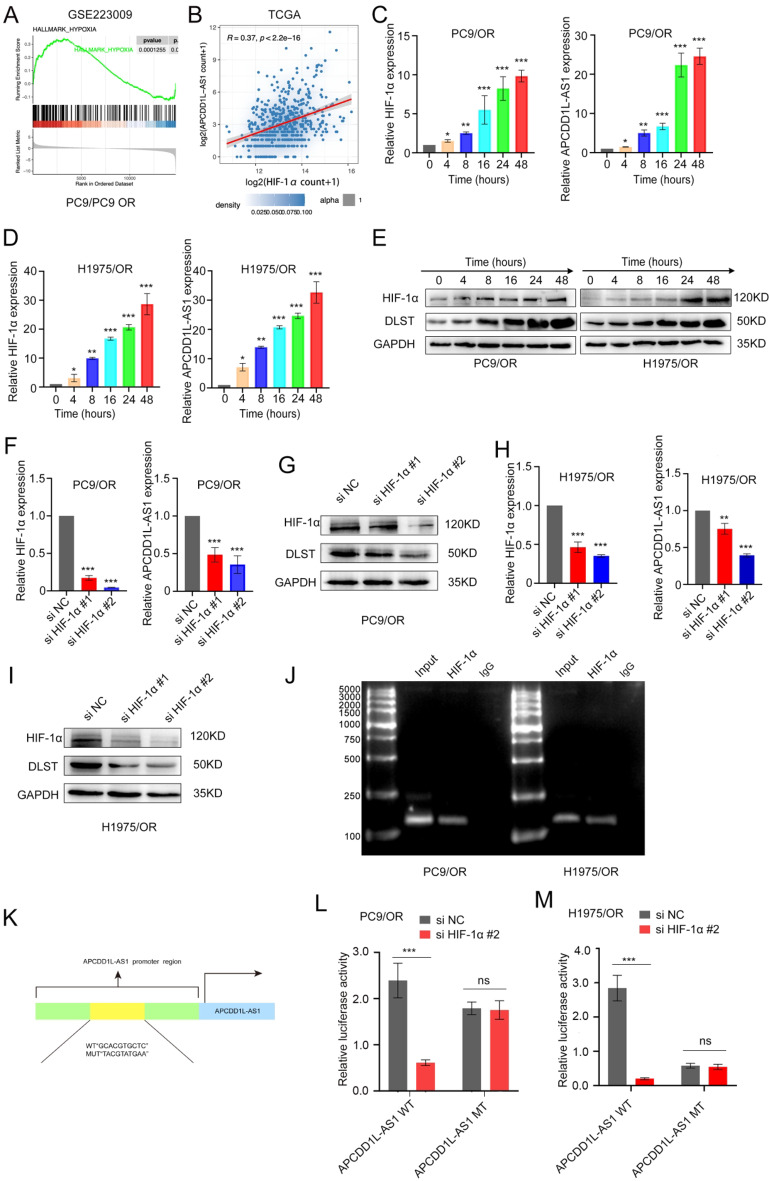
APCDD1L-AS1 is transcriptionally induced by HIF-1α under hypoxia. (**A**) Gene set enrichment analysis (GSEA) predicted that the genes that were highly co-expressed with APCDD1L-AS1 were enriched in “HYPOXIA”. (**B**) Correlation analysis showed that APCDD1L-AS1 levels were positively correlated with HIF-1α in TCGA LUAD tissues. (**C-E**) Hypoxic PC9/OR and H1975/OR cells treated with 1% oxygen showed increased expression of APCDD1L-AS1 and HIF-1α, according to qRT-PCR and western blot test. (**F-I**) QRT-PCR and Western blot assay demonstrated that silencing of HIF-1α down-regulated APCDD1L-AS1 expression. (**J**) ChIP assays showed that the DNA fragment containing the HIF-1α-binding site (+ 330/+339) was significantly enriched in the chromatin that was precipitated with an antibody against HIF-1α. (**K**) Two luciferase reporters constructed: APCDD1L-AS1 wild-typea and APCDD1L-AS1 mutation. (**L-M**) Dual-luciferase reporter assays demonstrated that HIF-1α silencing efficiently inhibited the luciferase activity of the wild-type group, but no significant change was observed in the mutation group. Data presented as mean ± standard error of mean (*n* = 3; statistical analysis by GraphPad Prism 8.0 (GraphPad Software Inc., San Diego, CA, USA); * *P* < 0.05; ** *P* < 0.01; *** *P* < 0.001)

Gene set enrichment analysis (GSEA) predicted that the genes enriched in “HYPOXIA” were highly co-expressed with APCDD1L-AS1 (Fig. 6A). Moreover, APCDD1L-AS1 levels were positively correlated with HIF-1α levels in the TCGA LUAD tissues (Fig. 6B). qRT-PCR and western blotting revealed that APCDD1L-AS1 and HIF-1α expression were elevated in hypoxic PC9/OR and H1975/OR cells following treatment with 1% oxygen (Fig. 6C-E). As shown in Fig. 6F-I, silencing of HIF-1α downregulated APCDD1L-AS1 expression under hypoxia in vitro. The JASPAR database was used to predict the HIF-1α-binding site sequences in the APCDD1L-AS1 promoter, and putative HIF-1α binding sites in the APCDD1L-AS1 promoter were located + 330 to + 339 bp upstream of the transcription start site. On the basis of these results, we hypothesised that HIF‑1α may transcriptionally activate APCDD1L-AS under hypoxia.

To determine whether APCDD1L-AS is a direct target of HIF-1α, we performed a dual-luciferase reporter and ChIP assays in PC9/OR and H1975/OR cells cultured under hypoxic conditions. As shown in Fig. 6J, the DNA fragment containing the HIF-1α-binding site (+ 330/+339) was significantly enriched in the chromatin that was precipitated with an antibody against HIF-1α. To perform the dual-luciferase reporter assay, we cloned two luciferase reporter constructs: pGL4.10-APCDD1L-AS1 wild-type, which included the APCDD1L-AS1 full-length promoter (− 2000 to 0 nt), and pGL4.10-APCDD1L-AS1 mutation, in which GCACGTGCTC (+ 330/+339) was replaced with TACGTATGAA. The results of dual-luciferase reporter assays demonstrated that HIF-1α silencing efficiently inhibited the luciferase activity of the wild-type group in comparison with the negative control group, but no significant change was observed in the mutation group in the PC9/OR and H1975/OR cells (Fig. 6L-M). These data confirm that HIF-1α can transcriptionally activate APCDD1L-AS1 through physical interaction with APCDD1L-AS1 promoter region under hypoxia.

### APCDD1L-AS1 targeting re-sensitises osimertinib-resistant LUAD cells to osimertinib and its expression positively correlates with that of DLST and HIF-1α in vivo

**Fig. 7:**
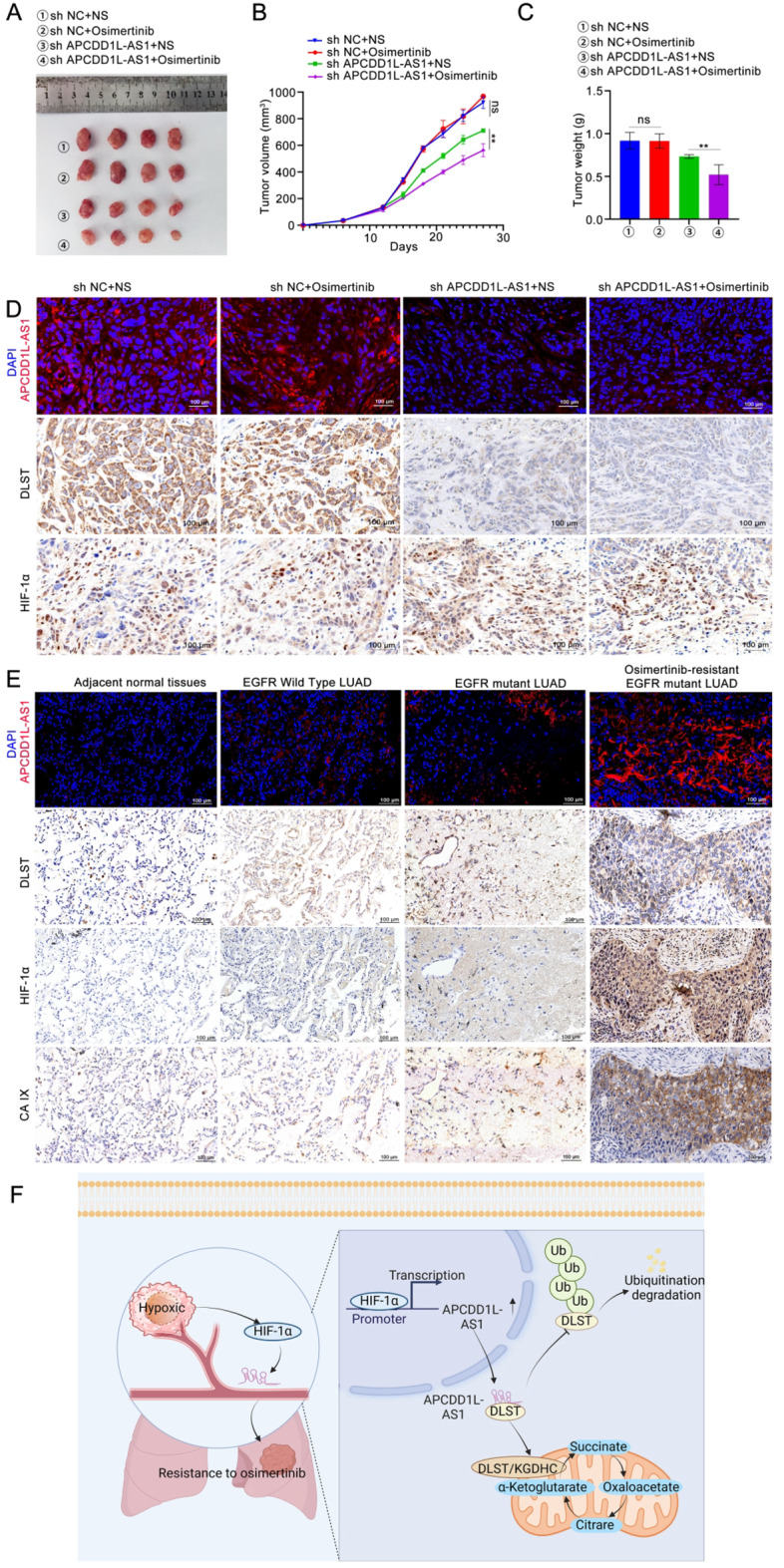
APCDD1L-AS1 targeting re-sensitizes osimertinib-resistant LUAD cells to osimertinib and its expression positively correlates with that of DLST and HIF-1α in vivo. (**A-C**) Tumor volume, weight, and growth curves of the subcutaneous tumor mouse models of PC9/OR cells infected with LV-sh APCDD1L-AS1 or LV-shNC treated with or without osimertinib (*n* = 4). (**D**) The expression levels of APCDD1L-AS1, DLST and HIF-α were detected by CISH and immunohistochemistry (IHC) analysis. (**E**) CISH and IHC analysis were used to analyze the expression of APCDD1L-AS1, DLST, HIF-α and CA IX in normal tissues, EGFR wild type LUAD, EGFR mutant LUAD, and osimertinib-resistant EGFR mutant LUAD samples. (**F**) A proposed working model for the mechanism by which APCDD1L-AS1 mediates its effect in LUAD resistance to osimertinib. Data presented as mean ± standard error of mean (*n* = 3; statistical analysis by GraphPad Prism 8.0 (GraphPad Software Inc., San Diego, CA, USA); * *P* < 0.05; ** *P* < 0.01; *** *P* < 0.001)

To explore the effect of APCDD1L-AS1 on osimertinib resistance in vivo, we constructed LVs carrying shNC and sh-APCDD1L-AS1, after estimating the transfection efficiency of lentiviral sh-APCDD1L-AS1 (Supplementary Fig. S9 A–B). PC9/OR cells were infected with LV-sh-APCDD1L-AS1 or LV-shNC and injected subcutaneously into BALB/c nude mice to establish a tumour xenotransplantation model. As shown in Fig. 7A-C, tumours in the APCDD1L-AS1-knockdown group were significantly smaller than those in the other groups after osimertinib treatment. Nodules in the LV-sh APCDD1L-AS1 plus osimertinib group exhibited the fewest Ki-67-positive cells (Supplementary Fig. S9 D). These data demonstrated that APCDD1L-AS1 targeting could re-sensitise osimertinib-resistant LUAD cells to osimertinib.

Moreover, the CISH and IHC analyses confirmed that in the internal tumour region, the APCDD1L-AS1-knockdown group exhibited remarkably fewer APCDD1L-AS1 and DLST- positive cells than the control group, while no significant change was observed in HIF-1α-positive cells (Fig. 7D). In the control group tumours, the levels of HIF-1α and APCDD1L-AS1 were significantly higher in the intratumoural regions than in the peripheral regions (Supplementary Fig. S9 E). Subsequently, IHC analyses revealed that the expression levels of HIF-1α and the hypoxic marker CA IX were significantly upregulated in LUAD tissues with osimertinib resistance in comparison with adjacent normal tissues (Fig. 7E). Kaplan–Meier analysis revealed that osimertinib-resistant LUAD patients with high CA IX expression (as determined by the median cutoff score) had worse OS than those with low CA IX expression (*P* = 0.037; Supplementary Fig. S9 F). This observation underscores the substantial correlation between hypoxia and the development of osimertinib resistance. Moreover, APCDD1L-AS1 expression was positively correlated with DLST, HIF-1α, and CA IX expression on the basis of CISH and IHC staining in osimertinib-resistant LUAD tissue samples (Fig. 7E& Supplementary Fig. S9 G). Collectively, these findings were consistent with our in vitro results, indicating that the HIF-1α**/**APCDD1L-AS1/DLST axis is involved in osimertinib resistance.

## Discussion

Osimertinib, a third-generation EGFR-TKI, is widely used as second-line treatment for patients with EGFR-acquired mutant (T790M) LUAD and as first-line treatment for EGFR-activating mutant (exon 19 or L858R deletion) LUAD [25]. Despite the outstanding performance of osimertinib in the treatment of LUAD, osimertinib resistance has become an inevitable issue, limiting long-term effects, and several EGFR-dependent and EGFR-independent mechanisms have been reported [26, 27]. Previous studies have suggested that EGFR-independent mechanisms are more important and common in osimertinib resistance owing to tumour heterogeneity [28]. Therefore, further elucidation of the mechanism of EGFR-independent osimertinib resistance is necessary to identify new potential therapeutic targets for overcoming osimertinib resistance. In the present study, we found that the lncRNA APCDD1L-AS1 was upregulated in osimertinib-resistant LUAD cells and tissues, and this upregulation was correlated with the poor survival of patients with osimertinib-resistant LUAD. In vitro and in vivo studies indicated that APCDD1L-AS1 induces osimertinib resistance in LUAD cells. Our data also demonstrated that APCDD1L-AS1 plays an important role in regulating the proliferation, invasion, and migration of PC9-P and H1975-P cells in vitro (Supplementary Fig. S4&S5). Mechanistically, APCDD1L-AS1 drives the TCA cycle in osimertinib-resistant LUAD cells by directly binding to and protecting DLST from ubiquitination and degradation. Moreover, we presented evidence that hypoxia-induced HIF-1α activates the transcription of APCDD1L-AS1 by binding to the APCDD1L-AS1 promoter region. Our data demonstrated that the HIF-1α/APCDD1L-AS1/DLST axis is a hitherto unidentified mechanism for conferring osimertinib resistance independent of EGFR mutations (Fig. 7F).

Hypoxia is a key microenvironmental stress factor associated with drug resistance in solid tumours [29]. Under hypoxic conditions, HIF-1α stabilises and accumulates in the nucleus to transcribe multiple genes involved in drug resistance [30]. Although hypoxia-induced HIF-1α has been suggested to cause osimertinib resistance in LUAD, the downstream mechanisms and multiple phenotypic changes associated with hypoxia-induced HIF-1α require elucidation [18, 29]. In the present study, we used GSEA, qRT-PCR, ChIP, and dual-luciferase reporter assays to show that APCDD1L-AS1 was transactivated by HIF-1α under hypoxic conditions, leading to osimertinib resistance in LUAD (Fig. 6). This observation indicates that silencing APCDD1L-AS1 could downregulate the expression of DLST without affecting the expression of HIF-1α, based on the CISH and IHC staining of xenograft tumour tissues (Fig. 7D). However, in regions with high HIF-1α expression, APCDD1L-AS1 expression was upregulated (Supplementary Fig. S9 E&G). Our findings from the in vitro and in vivo experimental results provide sufficient evidence to support our hypothesis that APCDD1L-AS1 is a hypoxia-induced gene regulated by HIF-1α. To our knowledge, this is the first report characterising APCDD1L-AS1 as a hypoxia-responsive lncRNA in osimertinib-resistant LUAD. Previous studies have shown that hypoxia limits energy production during glycolysis and suppresses the TCA cycle [31]. However, recent research has shown that cancer cells can dissociate glycolysis from the TCA cycle and rely heavily on the TCA cycle for energy production and macromolecular synthesis [32]. In this study, we confirmed that APCDD1L-AS1 directly interacts with DLST in the cytoplasm of osimertinib-resistant LUAD cells. Western blotting, qRT-PCR, and endogenous ubiquitination assays revealed that APCDD1L-AS1 increased DLST protein levels by protecting it from ubiquitin-proteasomal degradation, thereby activating the TCA cycle to confer osimertinib resistance in LUAD (Fig. 4). These results supported the notion that hypoxia-induced HIF-1α/APCDD1L-AS1/DLST axis reprogrammed the glycolysis and OXPHOS balance to confer osimertinib resistance in LUAD.

Overall, our results proved that hypoxia-induced HIF-1α upregulation of APCDD1L-AS1 expression confers osimertinib resistance in LUAD cells by reprogramming the TCA cycle. Targeting APCDD1L-AS1 significantly enhanced the susceptibility of PC9/OR cells to osimertinib *in vivo.* However, this study had some limitations. First, we did not design siRNA drugs to further evaluate the effect of targeting APCDD1L-AS1 in reversing osimertinib resistance in LUAD. Second, we did not address the mechanisms by which APCDDD1L-AS1 regulates the malignant progression of LUAD. Third, we used a relatively small number of clinical samples to evaluate the prognostic role of APCDDD1L-AS1 in osimertinib-resistant lung cancer. Fourth, APCDDD1L-AS1 may promote osimertinib resistance by regulating proteins other than DLST in lung adenocarcinoma, such as HSPA8; however, we did not further explore the mechanism. We will address these issues in future studies.

## Electronic supplementary material

Below is the link to the electronic supplementary material.

Supplementary Material 1

Supplementary Material 2

## Data Availability

No datasets were generated or analysed during the current study.

## Abbreviations

LUAD: Lung adenocarcinoma
EGFR: Epidermal growth factor receptor
TKI: Yrosine kinase inhibitor
lncRNAs: Long non-coding RNAs
TCA: The tricarboxylic acid
DLST: Dihydrolipoamide S-succinyltransferase
KGDHC: α-ketoglutarate dehydrogenase complex
OXPHOS: Oxidative phosphorylation
EdU: 5-Ethynyl-2’-deoxyuridine
CISH: Chromogenic in situ hybridization
OS: Overall survival
RIP: RNA immunoprecipitation
TCGA: The cancer genome atlas
GEO: Gene expression omnibus
HR: Hazard ratio
CI: Confidence interval
CHX: Cycloheximide
OCR: Oxygen consumption rates
GSEA: Gene set enrichment analysis
LV: Lentiviruses
IHC: Immunohistochemistry
Co-IP: Co-immunoprecipitation
ChIP: Chromatin immunoprecipitation
FISH: Fluorescence in situ hybridization
